# Effect of Plasticization/Annealing on Thermal, Dynamic Mechanical, and Rheological Properties of Poly(Lactic Acid)

**DOI:** 10.3390/polym16070974

**Published:** 2024-04-03

**Authors:** Lina Benkraled, Assia Zennaki, Latifa Zair, Khadidja Arabeche, Abdelkader Berrayah, Ana Barrera, Zohra Bouberka, Ulrich Maschke

**Affiliations:** 1 Laboratoire de Recherche sur les Macromolécules (LRM), Faculté des Sciences, Université Abou Bekr Belkaïd, BP 119, Tlemcen 13000, Algeria; 2 Unité Matériaux et Transformations (UMET), UMR 8207, Université de Lille, CNRS, INRAE, Centrale Lille, 59000 Lille, France; 3 Laboratoire Physico-Chimique des Matériaux, Catalyse et Environnement (LPCMCE), Université des Sciences et de la Technologie Mohammed Boudiaf d’Oran (USTO-MB), Oran 31000, Algeria

**Keywords:** poly(lactic acid), poly(ethylene glycol), plasticization, annealing, crystallinity

## Abstract

This study investigates the use of low molecular weight poly(ethylene glycol) (PEG) as a plasticizer for poly(lactic acid) (PLA). PLA/PEG blend films were prepared using the solvent casting method with varying mixing ratios. The films were analyzed using differential scanning calorimetry (DSC), dynamic mechanical analysis (DMA), and dynamic rheological analysis. The results indicate that the addition of PEG as a plasticizer affects the thermal and mechanical properties of the PLA/PEG blend films. The study found that the glass transition and cold crystallization temperatures decreased with increasing PEG content up to 20 wt%, while the crystallinity and crystallization rate increased. The blends with up to 20 wt% PEG were miscible, but phase separation occurred when the plasticizer content was increased to 30 wt%. Subsequently, amorphous samples of neat PLA and PLA plasticized with 10 wt% of PEG underwent annealing at various temperatures (*T*_a_ = 80–120 °C) for durations *t*_a_ of 1 and 24 h. The samples were then analyzed using DSC and DMA. The addition of PEG to PLA altered the content of α′ and α crystalline forms compared to neat PLA at a given (*T*_a_; *t*_a_) and favored the formation of a mixture of α′ and α crystals. The crystallinity achieved upon annealing increased with increasing *T*_a_ or *t*_a_ and with the incorporation of PEG.

## 1. Introduction

Recently, there has been an increasing interest in poly(lactic acid) (PLA) as a bio-based and biodegradable substitute for petroleum-based plastics [1,2]. PLA is derived from lactic acid through microbial fermentation of renewable resources, and it offers superior mechanical strength, transparency, and durability compared to other biodegradable plastics [3,4,5,6,7]. It shares properties with petroleum-based polymers and can be easily processed using common techniques [8,9,10]. Furthermore, PLA is approved for food contact and is compostable, bioresorbable, and biocompatible. This has led to its use in biomedicine, food packaging, and textiles [11,12,13].

Although PLA has desirable properties, it faces limitations such as brittleness, poor thermal stability, and slow crystallization kinetics, which hinder its industrial use [14,15,16]. Several strategies have been explored to address the limitations of PLA, including stereocomplexation, plasticization, filler incorporation, and polymer blending [1,13,17,18]. Plasticization is a cost-effective method for enhancing the flexibility of PLA by increasing free volume, reducing intermolecular forces, and improving molecular mobility [19,20,21]. Various plasticizers, including poly(ethylene glycol) (PEG) [22,23,24,25,26,27,28,29,30,31], poly(propylene glycol) (PPG) [32,33], oligomeric lactic acid (OLA) [6,34,35], citrate esters [36,37,38], and vegetable oils [39,40,41,42], have been utilized to blend with PLA. This blending process reduces PLA’s glass transition temperature and enhances its elongation at break and impact resistance. These findings offer valuable insights into expanding the industrial applications of PLA.

The impact of PEG as a plasticizer on the properties of PLA/PEG blends has been extensively studied [30,43]. PEG is highly miscible with PLA due to the close solubility parameters of the two materials and the reaction between the hydroxyl groups in PEG molecules and the carboxyl groups in PLA molecules. The miscibility of PEG with PLA is affected by its molecular weight and content [25,28,30]. Baiardo et al. [25] demonstrated that low molecular weight PEG offers the best performance in terms of miscibility and plasticizing efficiency. Li et al. [28] investigated the effects of PEG molecular weight and quantity on the thermal and mechanical properties of PLA/PEG blends. Their findings suggest that PEG-10,000 (*M*_w_ = 10,000 g/mol) significantly enhances the crystallization capacity and impact toughness of PLA. However, excessive PEG-10,000 content can result in phase separation due to crystallization.

Additionally, the process of plasticization has an impact on the crystallization behavior of PLA. Various plasticizers have been found to decrease the nucleation density, increase the rate of spherulitic growth, and affect the crystallinity and crystallization rate of PLA [32,40,42,44,45]. Greco et al. [42] found that the addition of PEG resulted in a significantly faster crystallization rate and a higher degree of crystallinity in PLA. The study emphasizes the significance of taking into account the molecular weight and quantity of PEG in plasticizing PLA and its effect on the properties of PLA/PEG blends.

In numerous applications, enhancing the crystallinity of polylactic acid (PLA) is desirable to improve its mechanical properties and heat resistance. However, the slow crystallization of PLA poses challenges in achieving significant crystallinity using common melt processing technologies. However, heating the material within a specific temperature range through annealing can significantly increase its crystallinity. Research has shown that annealing PLA at elevated temperatures for a certain duration results in a significant increase in crystallinity, leading to a notable improvement in heat deflection temperature [15,46].

PLA can crystallize into three forms: α, β, and γ, depending on the crystallization conditions. The research findings indicate that PLA can crystallize into both α and α′ forms, and the development of these forms is influenced by the crystallization temperature, time, and the molecular weight of PLA [46,47,48,49,50,51,52,53].

Furthermore, it was discovered that the melting behavior of PLA is affected by the crystallization temperature, specifically the α′ and α content. On the DSC thermograms, a minor exothermic peak was observed just before the melting peak when PLA was crystallized below 100 °C. This peak was associated with the α′-α (disorder-order) solid-state phase transition, without the melting of the α′ phase. However, the exotherm disappeared when PLA was crystallized between 100 °C and 120 °C, but the melting process became bimodal. The height of the first endotherm increased in relation to the second one as the crystallization temperature increased. Both endotherms were associated with the melting of the α phase. The first peak was attributed to the synchronous melting of the original α crystals and the α′-α phase transition, which occurred through a melt recrystallization mechanism. The second peak was attributed to the melting of the α crystals resulting from the α′-α transition. When the crystallization temperature exceeded 120 °C, only one melting peak was observed, which was related to the melting of the α phase. According to Tábi et al. [46], the content of the crystalline forms α′ and α significantly influenced the heat deflection temperature, mechanical properties, and creep resistance of PLA. These properties improved as the α to α′ ratio increased. Zennaki et al. [54] investigated the impact of annealing temperature (80–140 °C) and time (3–30 h) on the crystalline structure of PLA.

This study investigates the impact of using low molecular weight PEG (*M*_n_ = 400 g/mol) as a plasticizer for PLA. The study examines the effect of plasticization on the crystallization behavior, thermal, dynamic mechanical, and rheological properties of PLA. The miscibility of the PLA/PEG mixture is important in various applications. Its limits are defined and analyzed experimentally using DSC, DMA, and dynamic rheometry, as well as theoretically. Particular attention is also paid to correlating the crystalline forms α′ and α with crystallinity improvement. Additionally, the combined effect of PEG addition and annealing is assessed, with particular attention given to the crystallinity and crystalline structure of PLA.

## 2. Materials and Methods

### 2.1. Materials

The study utilized Ingeo 4043D PLA from NatureWorks (NatureWorks LLC, Minnetonka, MN, USA), a semi-crystalline film grade resin in pellet form containing approximately 4.5 mol% of D-isomer units. Sigma-Aldrich (Sigma-Aldrich, Saint-Quentin-Fallavier, France) provided liquid PEG-400, the plasticizer, while Honeywell (Honeywell, Seelze, Germany) supplied chloroform, the solvent.

### 2.2. Sample Preparation

PLA-based films with different PEG contents (1, 2, 5, 10, 15, 20, and 30 wt%) were prepared using the solvent casting method. To prevent hydrolytic degradation during processing, the highly hygroscopic PLA pellets were dried in a vacuum oven at 50 °C for 24 h before blending. PEG was used as received. PLA and PEG were dissolved separately in chloroform using an orbital shaker operating at 300–400 rpm under ambient conditions. The solutions were then mixed and stirred for approximately 3 h to form homogeneous solutions. The resulting solutions were poured into glass Petri dishes and allowed to evaporate at ambient temperature over a period of 3 days. Subsequently, the samples were dried under vacuum at 40 °C for 24 h to remove any residual traces of solvent. In order to obtain a reference material, neat PLA was also processed in the same manner. The resulting films were approximately 0.2 mm thick.

For the dynamic mechanical analysis, films were prepared with a thickness of approximately 0.25 mm from the solvent-casted samples. This was achieved by hot pressing at 190 °C, followed by immediate quenching into ice water (~2 °C) to erase the solvent history.

Annealing was performed on melt-quenched samples of neat PLA and PLA plasticized with 10% PEG (PLA/PEG-90/10). The samples underwent annealing in a vacuum oven at temperatures ranging from *T*_a_ = 80–120 °C for time intervals of *t*_a_ = 1 and 24 h. The annealed samples will be referred to as a-PLA (*T*_a_; *t*_a_) and a-PLA/PEG-90/10 (*T*_a_; *t*_a_) for PLA and PLA/PEG-90/10, respectively. The unannealed reference samples will be referred to as un-PLA and un-PLA/PEG-90/10. All materials were stored in a desiccator at ambient temperature until analysis.

### 2.3. Characterization

#### 2.3.1. Differential Scanning Calorimetry (DSC)

DSC measurements were conducted using a TA Instruments Q2000 differential scanning calorimeter (TA Instruments, New Castle, DE, USA) that was equipped with a refrigerated cooling system (RCS90). The temperature and heat flow scales of the calorimeter were calibrated using high purity indium standards. The scans were performed on 10–12 mg samples that were hermetically sealed into aluminum Tzero pans (TA Instruments, New Castle, DE, USA), under a nitrogen gas flow of 50 mL/min. The solvent-casted samples were heated to 180 °C for 3 min to erase the solvent history. They were then cooled to −60 °C and held for 2 min before being reheated to 180 °C. The heating and cooling rates were 10 and 20 °C/min, respectively. The annealed samples were heated from −60 to 180 °C at a rate of 10 °C/min.

#### 2.3.2. Dynamic Mechanical Analysis (DMA)

DMA measurements were performed using a TA Instruments Q800 dynamic mechanical analyzer (TA Instruments, New Castle, DE, USA) in tension mode. The oscillation amplitude and preload force were set to 15 μm and 0.01 N, respectively. The experiments were conducted from room temperature to 180 °C at a heating rate of 3 °C/min and a frequency of 1 Hz. The samples were rectangular with dimensions of approximately 25 × 5 × 0.25 mm^3^, and the span length was approximately 8 mm.

#### 2.3.3. Dynamic Rheological Measurements

Dynamic rheometry involves applying a sinusoidal shear solicitation to a material and measuring the resultant response. Controlled-strain rheometers induce a sinusoidal strain $\gamma (t)=\gamma_{0}\sin(\omega t)$ of low amplitude $\gamma_{0}$ (low enough to remain within the linear viscoelastic region of the material) and angular frequency *ω*, and measure the resulting sinusoidal stress $\sigma \left(t\right)=\sigma_{0}\sin(\omega t+\delta )$ of amplitude $\sigma_{0}$, with the same angular frequency *ω* but out of phase by a phase angle *δ* (0 < *δ* < π/2 for a viscoelastic material). Using complex notation for the strain $\gamma *(t)=\gamma_{0}e^{i\omega t}$ and the stress $\sigma *(t)=\sigma_{0}e^{i(\omega t+\delta )}$, the complex modulus is then defined as:

(1) $$G*(\omega )=\frac{\sigma *}{\gamma *}=\frac{\sigma_{0}}{\gamma_{0}}e^{i\delta }=\frac{\sigma_{0}}{\gamma_{0}}(\cos\delta +i\sin\delta )=G\prime (\omega )+iG\prime \prime \left(\omega \right)$$

with:

$$G\prime (\omega )=\frac{\sigma_{0}}{\gamma_{0}}\cos\delta \;\mathrm{and}\;G\prime \prime \left(\omega \right)=\frac{\sigma_{0}}{\gamma_{0}}\sin\delta$$

The storage (elastic) modulus *G*′(*ω*) characterize the energy stored by the material, and the loss (viscous) modulus *G*″(*ω*) characterize the energy dissipated by the material.

The complex viscosity η*(*ω*) is defined as:

(2) $$\eta *(\omega )=\frac{\sigma *}{\dot{\gamma }*}=\frac{\sigma_{0}}{\gamma_{0}i\omega }e^{i\delta }=\frac{\sigma_{0}}{\gamma_{0}}\frac{\sin\delta -i\cos\delta }{\omega }=\frac{G\prime \prime \left(\omega \right)}{\omega }-i\frac{G\prime (\omega )}{\omega }=\eta \prime (\omega )-i\eta \prime \prime \left(\omega \right)$$

with:

$$\left|\eta *(\omega )\right|=\sqrt{{\eta \prime }^{2}\left(\omega \right)+\eta {\prime \prime }^{2}\left(\omega \right)}=\frac{1}{\omega }\sqrt{G{\prime \prime }^{2}\left(\omega \right)+{G\prime }^{2}\left(\omega \right)}$$

To broaden the empirical Cole-Cole model spectrum, which was initially introduced in the context of dielectric relaxation, to η*(*ω*), Havriliak and Negami [55] proposed a generalization of the Cole–Cole equation as follows:

(3) $$\eta *(\omega )={\eta *}_{0}{[\;1+{\left(i\lambda \omega \right)}^{a}]}^{\frac{n-1}{a}}$$

The original phenomenological Carreau–Yasuda model which was developed for steady-state viscosity (i.e., $\eta (\dot{\gamma })$), is given by:

(4) $$\eta (\dot{\gamma })=\eta_{0}{[1+{\left(\lambda \dot{\gamma }\right)}^{a}]}^{\frac{n-1}{a}}$$

where $\eta_{0}$ is the zero-shear viscosity, *λ* represents the relaxation time which corresponds to the frequency of the onset of the shear thinning behavior, *a* stands for the Yasuda parameter which indicates the width of the transition region between the Newtonian and shear-thinning behavior and *n* corresponds to the power law index.

Within the linear viscoelastic regime, the applicability of the Cox–Merz rule (($\eta (\dot{\gamma })=\eta (\omega )$) for $(\dot{\gamma }=\omega )$) to linear polymers [56,57], allows to use the Carreau–Yasuda model for complex viscosity:

(5) $$\left|\eta *(\omega )\right|={\eta *}_{0}{[\;1+{\left(\lambda \omega \right)}^{a}]}^{\frac{n-1}{a}}$$

Lertwimolnun et al. [58] and Berzin et al. [59] extended the Carreau–Yasuda model for materials presenting an increase in viscosity at low frequency, adding the term *σ*_e_/*ω*, as follows:

(6) $$\left|\eta *(\omega )\right|=\frac{\sigma_{e}}{\omega }+{\eta *}_{0}{[\;1+{\left(\lambda \omega \right)}^{a}]}^{\frac{n-1}{a}}$$

where *σ*_e_ is the yield stress. The term *σ*_e_/*ω* is analogous to *σ*_e_/$\dot{\gamma }$ according to the Cox–Merz rule, where a strong increase in viscosity is observed towards low shear rates describing the elastic character of liquids, and often represented by Bingham behavior.

The rheological properties of the solvent-casted samples were measured using a TA Instruments Discovery Hybrid Rheometer (DHR-2) (TA Instruments, New Castle, DE, USA) with a 25 mm parallel plate geometry. The experiments were performed at 180 °C, and the samples were allowed to equilibrate for 1 min before measurements. The gap between the parallel plates was set to 0.2 mm for all tests. The linear viscoelastic region was determined by running strain sweep tests from 0.1 to 100% at an angular frequency of 10 rad/s. Frequency sweep tests were conducted subsequently at the selected strain of 5% over an angular frequency range of 600–0.1 rad/s.

## 3. Results and Discussion

### 3.1. Plasticization

#### 3.1.1. Thermal Behavior and Miscibility

**Figure 1:**
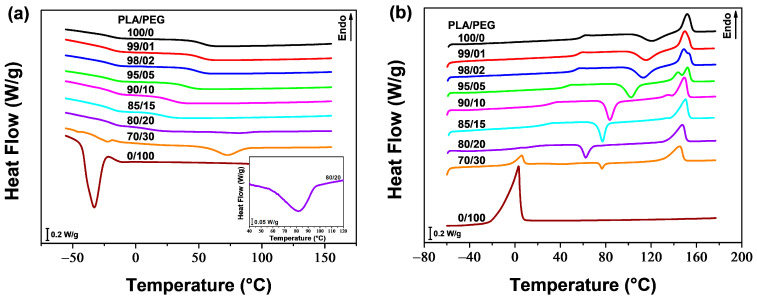
DSC thermograms of PLA/PEG blends during (**a**) cooling and (**b**) second heating.

The thermal properties of neat PLA, neat PEG, and their blends were analyzed using DSC. Figure 1a,b show the DSC thermograms recorded during cooling and second heating, respectively. The values of the glass transition temperature (*T*_g_), the cold crystallization temperature (*T*_cc_), the melting temperatures (*T*_m1_ and *T*_m2_) as well as the cold crystallization enthalpy (Δ*H*_cc_) and the melting enthalpy (Δ*H*_m_), were determined from the second heating scan. Table 1 displays the corresponding results, including the calculated crystallinity degree (*X*_c_) using Equation (7).

(7) $$X_{c}\;(\%)=\frac{{\Delta H}_{m}}{w_{\mathrm{PLA}}.{\Delta H}_{m}^{0}}\times 100$$

where $w_{\mathrm{PLA}}$ is the weight fraction of PLA in the blend and ${\Delta H}_{m}^{0}$ represents the enthalpy of fusion of fully crystalline PLA, which was reported by Fischer et al. [60] to be 93.1 J/g.

Figure 1a shows that neat PLA and PLA plasticized with 1, 2, 5, 10, and 15 wt% of PEG did not exhibit any crystallization peak during cooling from the melt (at a cooling rate of 20 °C/min). However, a weak and broad crystallization peak of the PLA phase was observed for PLA/PEG-80/20 near 82 °C (see insert in Figure 1a), and a clear and well-defined peak for PLA/PEG-70/30 at around 73 °C. Upon cooling, neat PEG demonstrated a strong ability to crystallize, with a distinct peak at approximately −33 °C. In addition to the PLA phase’s crystallization peak, a small peak was detected at around −22 °C for the blend containing 30 wt% of PEG, indicating phase separation at this concentration. Li et al. [28] found similar behavior when plasticizing PLA with PEG-10,000. They observed crystallization peaks of the PEG phase in the DSC cooling thermograms when the plasticizer content exceeded 10 wt%. This suggests a phase separation, which was further confirmed by means of transmission electron microscopy.

The thermograms of the second heating scan for neat PLA and PLA plasticized with up to 20 wt% of PEG (Figure 1b) show the cold crystallization of the PLA phase above the glass transition, followed by the melting of PLA crystals at higher temperatures (130–160 °C). PLA plasticized with 1, 2, 5, 10, and 15 wt% of PEG exhibit a double melting peak, with the lower temperature peak gradually fading away as the plasticizer content increases and the higher temperature peak becomes dominant. The first peak corresponds to the melting of the original α-form crystals that developed during non-isothermal cold crystallization. The second peak is associated with the melting of the disordered α′-form, which transformed to the ordered α-form through a melt-recrystallization process [46,53,61]. It is noteworthy that increasing the plasticizer content from 1 to 15 wt% leads to a decrease in the α to α′ ratio. However, when the PEG content reached 20 wt% and beyond, only one melting peak was observed, indicating the formation of α-form crystals. This double melting behavior of PLA when a plasticizer is added has been reported by several authors [6,14,26,62].

Figure 1b and Table 1 demonstrate that PLA samples containing up to 20 wt% of PEG exhibit a single *T*_g_. This *T*_g_ decreases with increasing plasticizer content, from 59.3 °C for neat PLA to 16.5 °C for PLA/PEG-80/20 (i.e., a decrease of 43 °C), indicating the miscibility of the blends. Similarly, *T*_cc_ decreases with increasing PEG content up to 20 wt%. The cold crystallization peak of neat PLA is centered at 121.5 °C, while those of plasticized samples become sharper and shift to lower temperatures. The gradual decrease in *T*_cc_ as PEG concentration increases, reaching 62.3 °C for PLA/PEG-80/20 (i.e., a decrease of 58 °C), indicates miscibility of the PLA/PEG systems up to 20 wt% of PEG content. The melting temperatures, however, did not significantly change with plasticizer content, but all blends exhibited a lower *T*_m1_ value compared to neat PLA.

A small crystallization peak was observed during the second heating scan for the blend containing 30 wt% of PEG. This is because the majority of crystallization occurred during the cooling process from the melt. The *T*_cc_ of this blend did not follow the same trend as the ones of PLA/PEG blends with lower PEG content, and increased to 76.7 °C. The DSC heating thermogram showed two distinct endothermic peaks for this sample. These peaks correspond to the melting peaks of the PEG phase and the PLA phase, respectively, confirming the phase separation of the blend. The *T*_g_, however, was not clearly defined due to the overlap of the PLA glass transition with the PEG melting peak.

**Figure 2:**
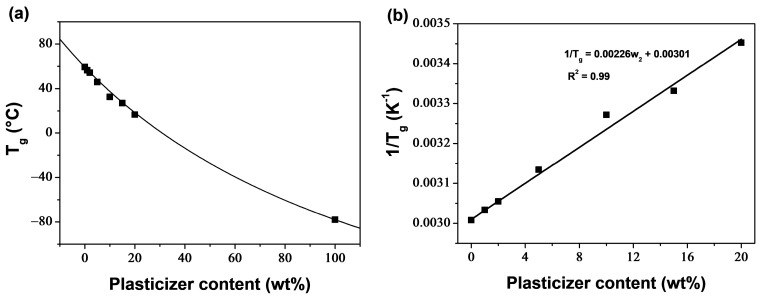
(**a**) Glass transition temperature of PLA/PEG blends: experimental data (symbols) and Fox equation (solid line). (**b**) Reciprocal of the glass transition temperature of PLA/PEG blends; the solid line represents the linear regression curve.

The Fox formula [63] was used to confirm the miscibility of mixtures containing up to 20 wt% of PEG.

(8) $$\frac{1}{T_{g}}=\frac{w_{1}}{T_{g1}}+\frac{w_{2}}{T_{g2}}$$

where *T*_g_ is the glass transition temperature of the blends, *T*_g1_ and *T*_g2_ are those of the blends’ components, and *w*_1_ and *w*_2_ are their weight fractions (with *w*_1_ + *w*_2_ = 1). Subscripts 1 and 2 refer to PLA and PEG, respectively. The *T*_g2_ of PEG could not be determined within the range of our DSC experiments; however, a value of −78 °C was reported for PEG-400 in the literature [64]. Figure 2a shows the application of the Fox equation to the *T*_g_s measured by DSC for PLA/PEG blends. The Fox equation was constructed using the measured *T*_g_ of PLA (*T*_g1_ = 59.3 °C) and the *T*_g_ reported in the literature for PEG 400 (*T*_g2_ = −78 °C). The measured *T*_g_s conform well to the Fox equation, indicating that the PLA/PEG blends in this study are miscible up to at least 20 wt% of PEG content.

Additionally, miscibility can be examined without the need for the *T*_g_ of PEG. In fact, Equation (8) can be rewritten as:

(9) $$\frac{1}{T_{g}}=\left(\frac{1}{T_{g2}}-\frac{1}{T_{g1}}\right)w_{2}+\frac{1}{T_{g1}}$$

Equation (9) shows that 1/*T*_g_ has a linear relationship with *w*_2_, indicating miscibility when the reciprocals of the measured *T*_g_s vary linearly with the plasticizer content. Figure 2b demonstrates good linearity between the reciprocals of *T*_g_s and the change in PEG content (*R*^2^ = 0.99), confirming miscibility within the plasticizer concentration range of 0–20 wt%. Furthermore, the *T*_g_ of PEG can be estimated from the slope of the linear regression curve, and was found to be −83.4 °C, slightly lower than the experimental value reported in the literature (−78 °C).

**Figure 3:**
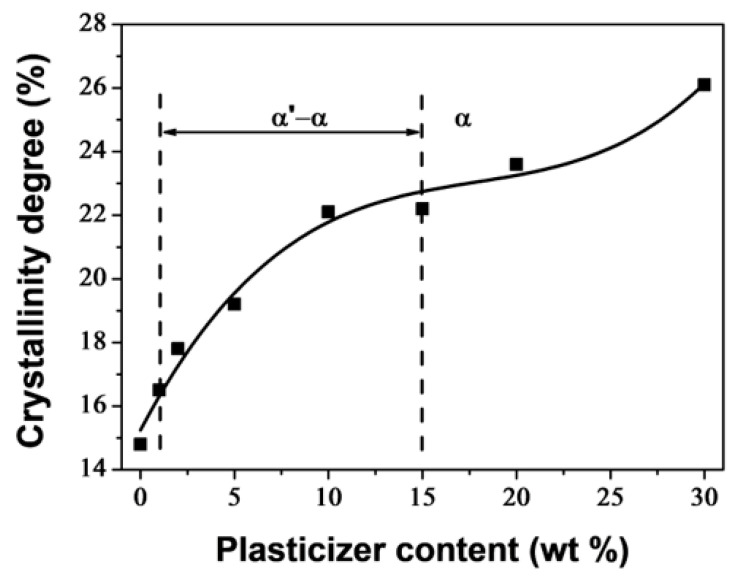
Crystallinity degree of PLA/PEG blends as function of PEG content.

The degree of crystallinity (*X*_c_), calculated using Equation (7), increased with higher PEG content, as shown in Figure 3. The plasticizer enhanced the crystallization ability of PLA by increasing polymer chain mobility. In the concentration range of 1–15 wt% of plasticizer, there was a significant increase in *X*_c_, from 14.8 wt% for neat PLA to 22.2 wt% for PLA/PEG-90/15. An inflection point was noticed at 15 wt% of PEG content, and for high PEG content, *X*_c_ continued to increase. The increase in crystallinity in the plasticizer content range of 1–15 wt% was accompanied by a decrease in the α to α′ ratio, but outside this range, only the α crystals developed. PLA/PEG-70/30 reached the highest value of *X*_c_, which was of 26.1%, indicating evidence of phase separation.

The decrease of *T*_g_ and *T*_cc_, along with the increase in *X*_c_ as the plasticizer level increased, were attributed to the improved molecular chain mobility of PLA resulting from the lubricating effect of PEG. The molecules of PEG diffused into the PLA matrix, penetrating between the polymer chains. This increased the free volume and decreased the intermolecular polymer chain interactions, resulting in improved chain mobility at lower temperatures [20,21].

#### 3.1.2. Non-Isothermal Cold Crystallization Kinetics

Crystallization is a first order phase transition in which a material undergoes a transition from an amorphous state to a crystalline phase. The crystallization enthalpy can be determined by measuring the area under the exothermic peak during the crystallization process. The equation for expressing the relative crystallinity (*X_T_*) as a function of temperature is as follows [65]:

(10) $$X_{T}=\frac{\int_{T_{0}}^{T}(\frac{{dH}_{\mathrm{cc}}}{dt})dt}{\Delta H_{\mathrm{cc}}}$$

where *T*_0_ is the temperature at the crystallization onset, *T* represents a temperature during the crystallization process, d*H*_cc_ corresponds to the enthalpy of crystallization released during an infinitesimal temperature range d*t*, and Δ*H*_cc_ stands for the overall heat released during the crystallization process.

Assuming minimal thermal lag between the sample and the DSC furnace, the relationship between crystallization time t and sample temperature T can be formulated as follows [66]:

(11) $$t=\frac{T-T_{0}}{\phi }$$

where *ϕ* represents the heating rate.

**Figure 4:**
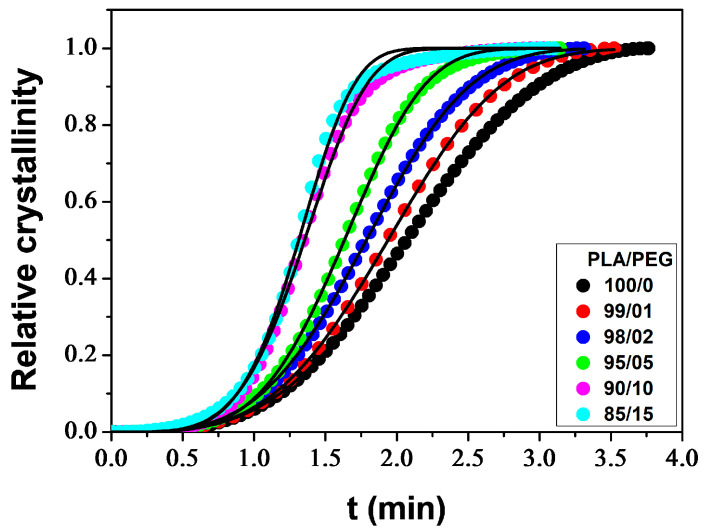
Relative crystallinity as function of time for PLA/PEG blends at a heating rate of 10 °C/min: experimental data (symbols) and Avrami model (solid line).

Figure 4 shows the change in relative crystallinity over time for PLA/PEG blends that were non-isothermally cold crystallized at a heating rate of 10 °C/min, with plasticizer content ranging from 0–15 wt%. All curves have a sigmoidal shape. The plateau of the curves in the early stage of crystallization reflects the induction period during which nuclei were formed. Subsequently, the crystals grew, as evidenced by the ascending part of the curves. During the final stage of crystallization, the upward curvature of the plots is attributed to secondary crystallization resulting from the impingement of spherulites. The crystallization rate decreased in this stage, but the crystallinity continued to increase slowly until the completion of the crystallization process [67,68].

Several models have been proposed in the literature for the quantitative description of crystallization kinetics, the most common approach being that of Avrami [69]:

(12) $$X_{t}=1-\exp(-{kt}^{n})$$

where *X_t_* stands for the relative crystallinity depending on time *t*, *k* is the crystallization rate constant depending on the nucleation and growth rate, and *n* represents the Avrami exponent depending on the nucleation type and the growth geometry of the crystals. The Avrami equation was proposed for isothermal crystallization. For non-isothermal processes, the parameter *k* should be appropriately corrected because the temperature constantly changes during the measurements, affecting the rates of both nuclei formation and spherulite growth, which are temperature-dependent [45]. Assuming a constant heating rate (*ϕ*), Jeziorny [70] proposed a correction for the crystallization rate constant as follows:

(13) $$\log k=\log\left(\frac{k_{c}}{\phi }\right)$$

where *k*_c_ is the corrected crystallization rate constant. The study utilized the Avrami equation to conduct an analysis by fitting the experimental *X_t_* data to Equation (12), as illustrated in Figure 4. Table 2 lists the values of the kinetic parameters (*n*, *k* and *k*_c_) along with the *R*^2^ parameter. The Avrami method offers a suitable description of the non-isothermal crystallization for the samples under study. However, minor deviations were observed at high *X_t_* (>90%) due to the neglect of the significant role of the secondary crystallization process [45]. Table 2 shows that *n* values increase with higher plasticizer content, indicating that the addition of PEG affects the mechanisms of nucleation and growth of PLA crystals. The *n* parameter ranges from 3.34 to 4.73, suggesting three-dimensional spherulitic growth with sporadic or simultaneous nucleation types [71]. The *k*_c_ parameter increases with increasing plasticizer content, indicating that the crystallization process of PLA was accelerated by the addition of PEG. This phenomenon was also observed in PLA plasticized with jojoba oil [45] and PLA plasticized with thermoplastic starch [72]. The plasticizer enhances the chain mobility of PLA, which in turn enhances the crystallization rate by reducing the energy required for the chain folding process during crystallization.

An important kinetic parameter, the crystallization half-time (*t*_1/2_), defined as the time required to reach half crystallinity (*X_t_* = 0.5), can be calculated from the Avrami parameters as follows:

(14) $$t_{1/2}={\left(\frac{\ln2}{k}\right)}^{1/n}$$

The values of *t*_1/2_ obtained, as shown in Table 2, decrease with increasing plasticizer level. This demonstrates that the crystallization rate increased as the plasticizer content increased.

#### 3.1.3. Dynamic Mechanical Behavior

**Figure 5:**
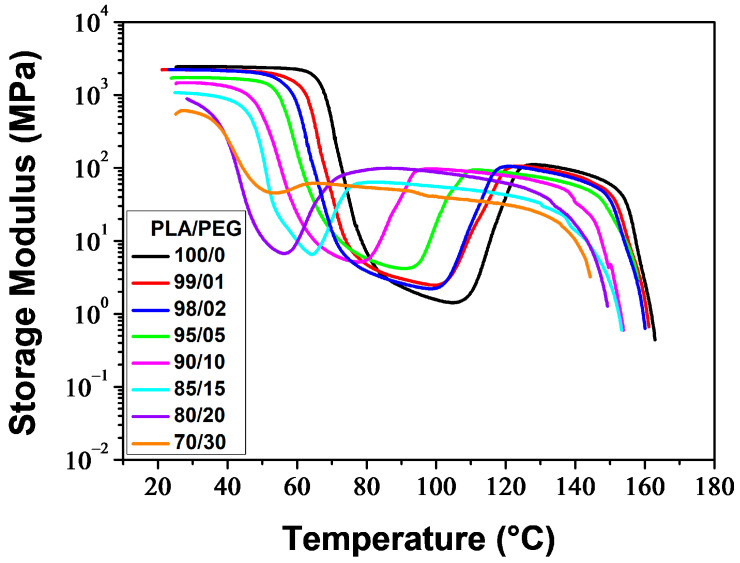
Storage modulus of neat and plasticized PLA.

**Figure 6:**
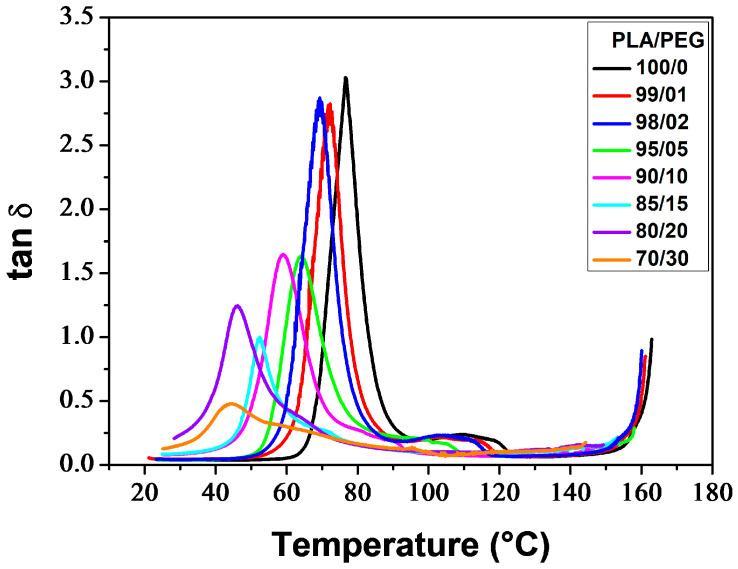
tan *δ* of neat and plasticized PLA.

Figure 5 and Figure 6 illustrate the temperature dependence of the storage modulus (*E*′) and loss factor (tan *δ*) for the melt-quenched PLA/PEG blends. At low temperatures, *E*′ displayed a glassy plateau (*E*_g_), characterizing the glassy rigid state. As the temperature increased, *E*′ decreased as the samples entered the glass transition region. The decrease of *E*′ was observed at lower temperatures as the PEG content increased. Both neat PLA and PLA plasticized with up to 20 wt% of PEG showed a significant decrease in *E*′ (two-three orders of magnitude) to a minimum value of ${E\prime }_{\min}$, followed by a sharp increase due to cold crystallization. After the glass transition, PLA chains became mobile enough to form crystalline regions, which was facilitated by a slow heating rate (3 °C/min) and sinusoidal solicitation. The temperature at which *E*′ starts to increase is considered the temperature of the onset of crystallization (*T*_cc′_). The significant decrease of *E*′ in the glass transition region, followed by the sharp increase resulting from the cold crystallization for the compositions containing up to 20 wt% of PEG, indicates the amorphous state of these materials before analysis. The blend containing 30 wt% of PEG exhibited a reduced decline of *E*′ in the glass transition region, followed by a slight increase. This suggests that most of the crystallization had already occurred before analysis and the sample was not amorphous. The crystallization caused a stiffening of the macromolecular chains, resulting in an increase of *E*′ up to a rubbery plateau (*E*_N_), which characterizes the rubbery elastic state. The value and length of the rubbery plateau depend on the PEG content. At higher temperatures, *E*′ decreased as the materials began to flow due to the melting of crystals.

One notable observation from *E*′ versus temperature curves is that the rubbery plateau expands as the PEG level increases. This means that the rubbery elastic state is reached more quickly with higher plasticizer content. Another significant effect is the reduction in the size of the hollow formed in the glass transition and cold crystallization regions (due to the decrease and increase of *E*′), as the PEG content increases. Additionally, the hollow shifts towards lower temperatures with a decrease in *T*_cc′_, which is attributed to the improvement of PLA chain mobility. Kang et al. [41] reported similar behavior for PLA plasticized with cardanol (CD). They observed that as the CD content increased up to 15 wt%, *E′* gradually decreased at the glassy state and the temperature at which *E′* began to rise shifted to a lower temperature. This indicates that the introduction of CD enhanced the cold crystallization ability of PLA.

Figure 6 thermograms illustrate the α-relaxation process, which is associated with the glass transition, for neat and plasticized PLA. The glass transition temperature (*T*_α_), determined at the maximum of the tan *δ* peak, decreased with increasing plasticizer level. Additionally, the incorporation of PEG resulted in lower peaks. PLA/PEG-70/30 exhibited a broad and low-intensity peak, which was observed due to crystallization before analysis. The tan *δ* curves also displayed bumps after the glass transition peaks (for low PEG content) or shoulders on the downward side of the peaks (for high PEG content), which were associated with the cold-crystallization process [73,74].

Table 3 reports the main thermo-mechanical properties resulting from the DMA thermograms shown in Figure 5 and Figure 6. These properties include the glassy plateau modulus (*E*_g_), the rubbery plateau modulus (*E*_N_), the glass transition temperature (*T*_α_), the temperature of the onset of crystallization (*T*_cc′_), and the extent in temperature of the rubbery plateau (Δ*T*_N_).

**Figure 7:**
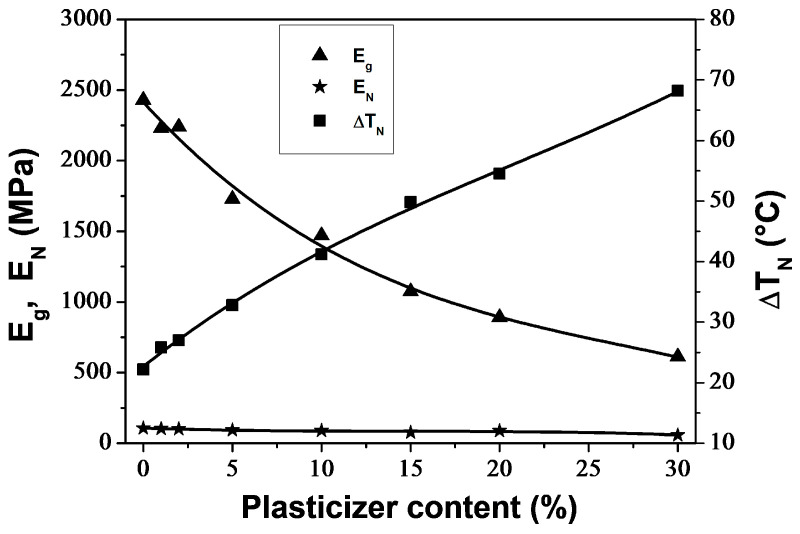
Glassy plateau modulus (*E*_g_), rubbery plateau modulus (*E*_N_) and extent in temperature of the rubbery plateau (Δ*T*_N_) for neat and plasticized PLA.

Figure 7 illustrates the impact of PEG content on *E*_g_, *E*_N_ and Δ*T*_N_. As the PEG content increased, the *E*_g_ of PLA/PEG blends decreased from 2428 MPa for neat PLA to 611 MPa for PLA/PEG-70/30, indicating a reduction in rigidity. The *E*_N_ values were relatively low and decreased slightly from 107 MPa for neat PLA to 58 MPa for PLA/PEG-70/30 with the addition of PEG. The rigidity of the materials in the rubbery plateau region came from the crystallites, which behaved like crosslinking nodes. Meanwhile, the Δ*T*_N_ significantly increased from 22.2 °C for neat PLA to 68.2 °C for PLA/PEG-70/30 due to the shift of *T*_cc′_ towards lower temperatures with increasing PEG content.

**Figure 8:**
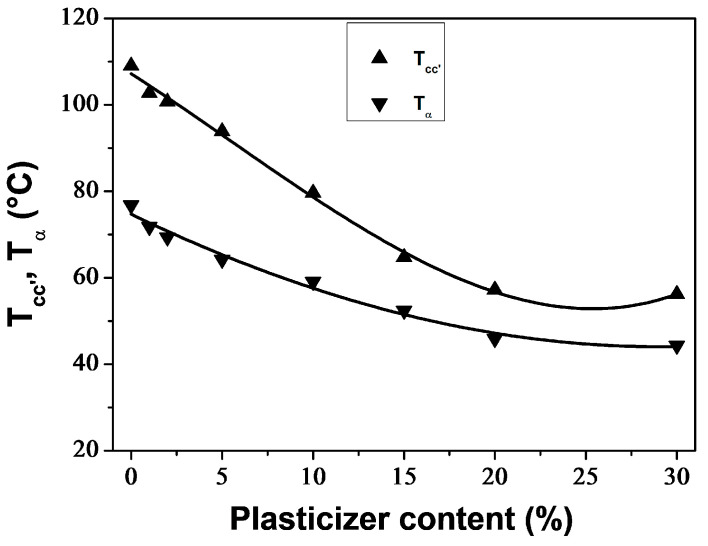
Glass transition temperature (*T*_α_) and temperature of the onset of crystallization (*T*_cc′_) for neat and plasticized PLA, obtained from DMA thermograms.

Figure 8 shows that increasing the plasticizer concentration decreased *T*_α_ and *T*_cc′_ in a monotonous manner until reaching a plateau value at around 20 wt% PEG content. This indicates phase separation in the blend containing 30 wt% PEG, as demonstrated by the presence of two melting peaks in the respective DSC thermogram. *T*_α_ and *T*_cc′_ exhibited similar trends to *T*_g_ and *T*_cc_ as determined by DSC technique (Table 1), but with *T*_α_ values approximately 15 °C higher than *T*_g_ values. Averous et al. [75] attribute the difference between the temperatures corresponding to the transitions observed by DMA and DSC to the frequency of the analysis method.

#### 3.1.4. Rheological Properties

The melt viscosity of polymers is highly sensitive to changes in the structure of macromolecular chains and the addition of plasticizers. Plasticizers, by definition, increase the free volume of the polymer and the mobility of the chains. Therefore, rheological measurements are both of practical and fundamental interest in this study. The study measured the storage modulus (*G*′), loss modulus (*G*″), and complex viscosity (η*) of PLA/PEG blends at a temperature of 180 °C and a strain of 5% over an angular frequency range of *ω* = 600–0.1 rad/s.

**Figure 9:**
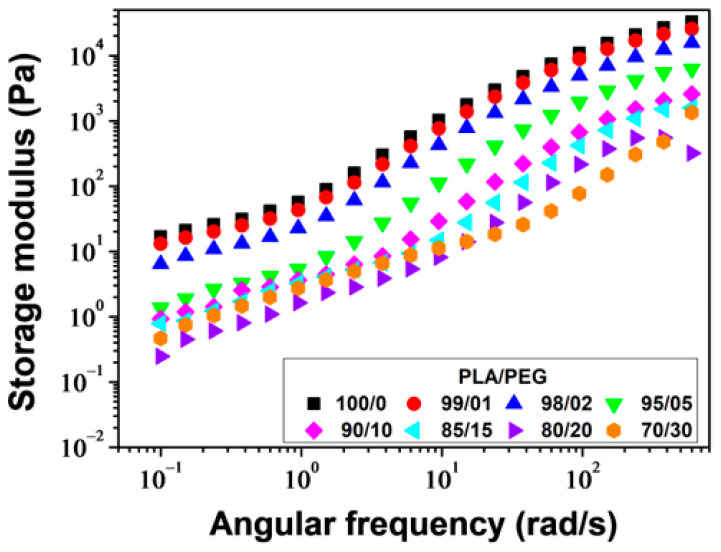
Storage modulus versus angular frequency for PLA/PEG blends at 180 °C.

Figure 9 shows the plots of *G*′ versus angular frequency for PLA/PEG blends. For all compositions, *G*′ increased as the angular frequency increased, but decreased with increasing PEG content. The decrease in *G*′ with increasing PEG content was due to a decrease in molecular entanglements. PLA samples plasticized with up to 20 wt% of PEG showed parallel log *G*′ versus log *ω* slopes beyond *ω* ≈ 2 rad/s. Below *ω* ≈ 2 rad/s, a decrease in the slopes was observed due to the re-entanglement of the molecular chains, resulting in excess elasticity. For PLA/PEG-70/30, a significant change in the slope occurred around *ω* ≈ 30 rad/s, due to a high rate of re-entanglement, which can be attributed to the phase separation of this mixture, as described in previous sections.

**Figure 10:**
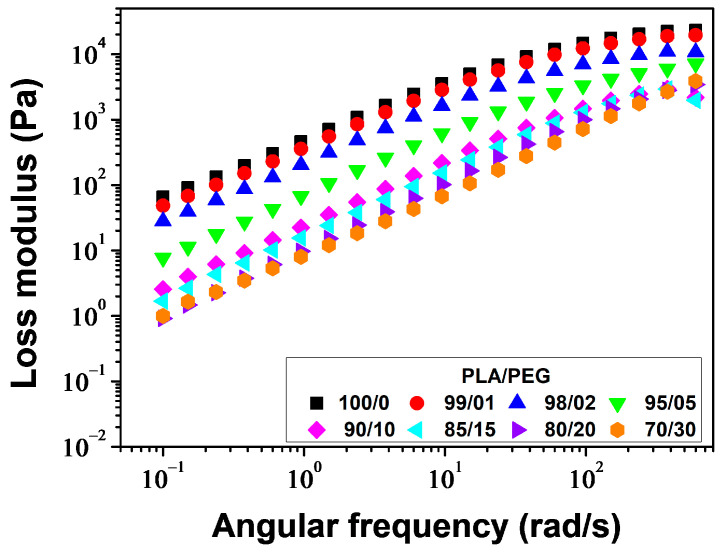
Loss modulus versus angular frequency for PLA/PEG blends at 180 °C.

Figure 10 displays the plots of *G*″ versus angular frequency. *G*″ increased linearly with increasing angular frequency for all samples but decreased with increasing PEG content. The decrease of *G*″ with increasing PEG concentration indicates a positive plasticization effect.

**Figure 11:**
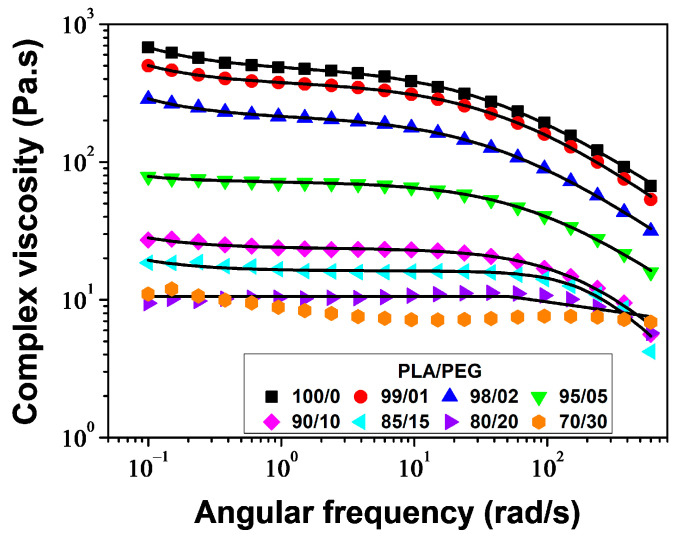
Complex viscosity versus angular frequency for PLA/PEG blends at 180 °C: experimental data (symbols) and the model of Carreau–Yasuda with yield stress (solid line).

Figure 11 shows the angular frequency dependence of complex viscosity (η*) for various samples. The samples containing 0–20 wt% of PEG exhibit a Newtonian plateau, followed by a shear thinning behavior above *ω* ≈ 30 rad/s. Neat PLA and PLA plasticized with 1 and 2 wt% of PEG exhibit an increase in viscosity with decreasing angular frequency below *ω* ≈ 1 rad/s. This behavior is described using the Carreau–Yasuda model with yield stress [58,59]:

(15) $$\eta *(\omega )=\frac{\sigma_{e}}{\omega }+{\eta *}_{0}{[\;1+{\left(\lambda \omega \right)}^{a}]}^{\frac{n-1}{a}}$$

where *σ*_e_ is the yield stress, ${\eta *}_{0}$ represents the zero-shear viscosity, *λ* stands for the relaxation time which corresponds to the frequency of the onset of the shear thinning behavior, *a* corresponds to the Yasuda parameter which indicates the width of the transition region between the Newtonian and shear-thinning behavior and *n* is the power law index. The values of these parameters, along with the *R*^2^ parameter, are presented in Table 4. The model accurately fit the complex viscosities of PLA/PEG blends containing up to 20 wt% of PEG. The plasticizing effect of PEG was demonstrated by a significant decrease in ${\eta *}_{0}$ from 490 to 10 Pa·s, *σ*_e_ from 19.5 to 0 Pa, and *λ* from 0.0251 to 0.0045 s. The curve of η* versus *ω* confirms the phase separation of PLA/PEG-70/30, as already demonstrated by DSC. It shows two relaxation times: the first between *ω* ≈ 0.1 and *ω* ≈ 9.5 rad/s with a relaxation time *λ*_1_ ≈ 2.6420 s, and the second between *ω* ≈ 9.5 and *ω* ≈ 230 rad/s with a relaxation time *λ*_2_ ≈ 0.0042 s. This observation indicates the presence of two distinct relaxation processes in the material, confirming the phase separation effect of PLA/PEG-70/30 already demonstrated by DSC.

### 3.2. Annealing

#### 3.2.1. Thermal Properties and Crystalline Structure

**Figure 12:**
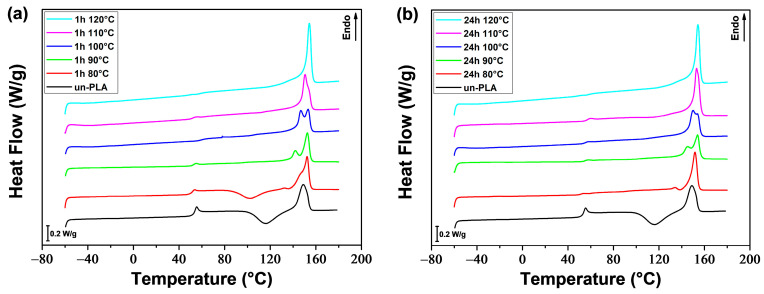
DSC thermograms of PLA samples annealed at various temperatures for (**a**) 1 h and (**b**) 24 h.

**Figure 13:**
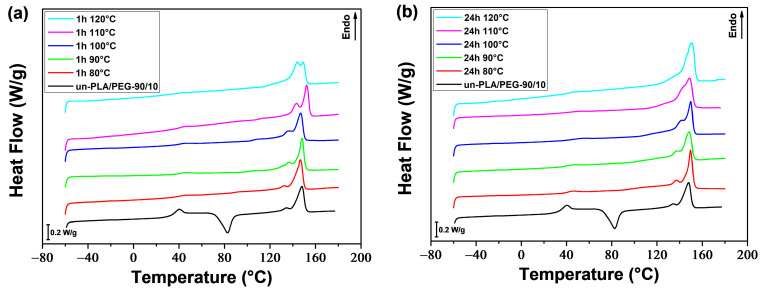
DSC thermograms of PLA/PEG-90/10 samples annealed at various temperatures for (**a**) 1 h and (**b**) 24 h.

The thermal properties and crystalline structure of PLA and PLA/PEG-90/10 samples were investigated by DSC after annealing at various temperatures (*T*_a_ = 80–120 °C) for different durations (*t*_a_ = 1 and 24 h). Figure 12 and Figure 13 show the DSC thermograms obtained during the first heating for a-PLA and a-PLA/PEG-90/10 samples, respectively. The DSC thermograms provided the thermal characteristics, which are listed in Table 5 and Table 6. The tables also list the degree of crystallinity (${X\prime }_{c}$), which is calculated using the following equation:

(16) $${X\prime }_{c}\;(\%)=\frac{{\Delta H}_{m}-{\Delta H}_{\mathrm{cc}}}{w_{\mathrm{PLA}}.{\Delta H}_{m}^{0}}\times 100$$

The reference samples that were melt-quenched (unannealed) showed cold-crystallization peaks at approximately 117 °C and 83 °C for un-PLA and un-PLA/PEG-90/10, respectively, due to their amorphous nature. In the annealed samples, the cold-crystallization peak was absent, except for a-PLA (*T*_a_ = 80 °C; *t*_a_ = 1 h), which showed a cold-crystallization peak at 103 °C. This indicates that the crystallization of this sample was incomplete during annealing (${X\prime }_{c}$ = 7.9%).

The melting behavior of PLA is significant as it provides insight into the crystalline structure formed during annealing. The shape of the melting peaks, and therefore the crystalline order of PLA (α and α′ content), was affected by the annealing time and temperature, as well as the addition of PEG.

The PLA samples annealed at 80 °C for 1 h and 24 h exhibited a small exothermic peak just prior to the melting peak related to the α′-α solid-state phase transition. This suggests that only the disordered α′ crystalline form developed during annealing, as previously reported and discussed by various authors [50,51,53]. By increasing the temperature (*T*_a_), the exothermic peak before melting disappeared, but instead, a double melting peak was observed for a-PLA samples when *T*_a_ was set to (*T*_a_ = 90–110 °C) for 1 h or (*T*_a_ = 90–100 °C) for 24 h. According to the literature [46,53,61,76], the double melting peak indicates that both the disordered α′ and ordered α crystals developed during annealing. The low-temperature endotherm is associated with the synchronous melting of the α crystals developed during annealing and the transformation of the α′ phase to α phase through a melt-recrystallization mechanism. The high-temperature endotherm is associated with the melting of the α crystals resulting from the α′-α transition. It is evident that increasing *T*_a_ or *t*_a_ leads to an increase in the first melting peak area over the second melting peak area, indicating a rise in the α phase content and a decrease in α′ phase content. The PLA samples annealed at higher temperatures (*T*_a_ = 120 °C; *t*_a_ = 1 h and *T*_a_ = 110 and 120 °C; *t*_a_ = 24 h), exhibited a single intense melting peak, indicating that only the ordered α form resulted from annealing.

The study found that neat PLA forms a mixture of α′ and α phases during annealing within a temperature range of 90 °C to 110 °C, as indicated by the double melting behavior. This result is consistent with the findings of Zennaki et al. [54]. Additionally, Zhang et al. [53] demonstrated that PLA with a molecular weight of *M*_w_ = 150,000 g/mol crystallizes isothermally from the melt and forms a mixture of α′ and α phases when the temperature is between 100 °C and 120 °C. Tábi et al. [46] investigated the crystalline structure of annealed PLA as a function of annealing temperature. They found that the specimens developed entirely α′, both α′ and α, or entirely α crystal structure when annealed at or below 100 °C, between 110–130 °C, or at or above 140 °C, respectively.

For a-PLA/PEG-90/10 samples, the small exotherm preceding the melting peak was not observed at any annealing temperature or time. However, a double melting peak was found for all samples except a-PLA/PEG-90/10 (*T*_a_ = 120 °C; *t*_a_ = 24 h), which showed a single melting peak. The addition of PEG to PLA resulted in the formation of a mixture of α′ and α crystals. The α to α′ ratio increased with increasing *T*_a_ or *t*_a_.

**Figure 14:**
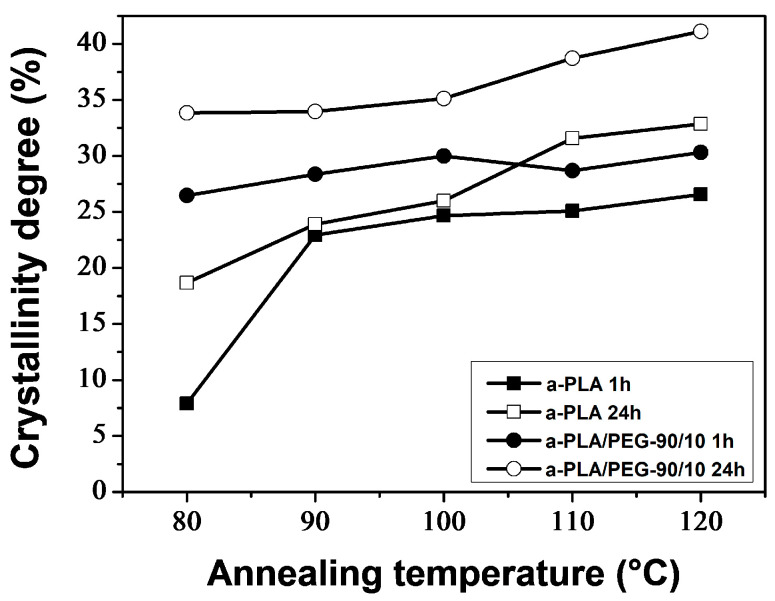
Crystallinity degree as function of annealing temperature for a-PLA and a-PLA/PEG-90/10 samples.

Figure 14 shows that the annealing time and temperature, as well as the addition of PEG, affected the crystallinity of PLA. The crystallinity increased with increasing *T*_a_ or *t*_a_ for both a-PLA and a-PLA/PEG-90/10 samples. Under the same annealing conditions (i.e., same *T*_a_ and *t*_a_), a-PLA/PEG-90/10 samples had higher crystallinity than a-PLA samples, and the difference in crystallinity was greater for *T*_a_ ≤ 100 °C. Although annealing for 1 h at 80 °C was not enough for a-PLA to complete crystallization, a-PLA/PEG-90/10 achieved full crystallization with over a three-fold increase in crystallinity. Additionally, for *T*_a_ temperatures ranging from 80–100 °C, the a-PLA/PEG-90/10 samples that were annealed for 1 h exhibited higher crystallinity compared to the a-PLA samples that were annealed for 24 h. The faster crystallization of plasticized PLA and higher crystallinity can be attributed to the improved PLA chain mobility resulting from the incorporation of PEG [42].

#### 3.2.2. Dynamic Mechanical Behavior

**Figure 15:**
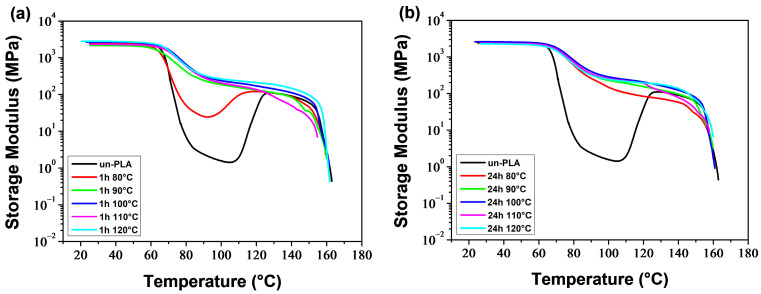
Storage modulus of PLA samples annealed at various temperatures for (**a**) 1 h and (**b**) 24 h.

**Figure 16:**
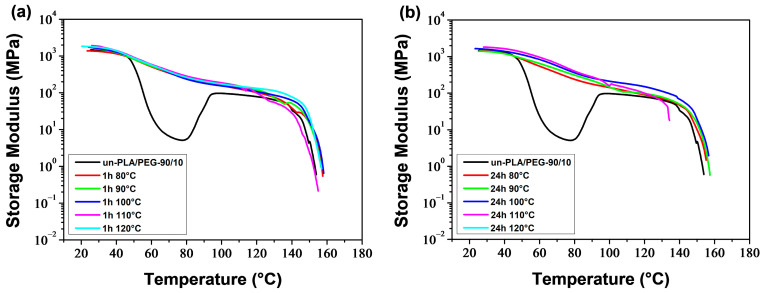
Storage modulus of PLA/PEG-90/10 samples annealed at various temperatures for (**a**) 1 h and (**b**) 24 h.

Figure 15 and Figure 16 display the temperature dependence of the storage modulus (*E′*) for the a-PLA and a-PLA/PEG-90/10 samples, respectively. The unannealed samples, which are amorphous, showed a significant decrease in *E′* in the glass transition region (60–105 °C and 40–77 °C for un-PLA and un-PLA/PEG-90/10, respectively), followed by a sharp increase due to cold crystallization [37]. After the glass transition, the PLA chains became mobile enough to form crystalline regions. This process was facilitated by a slow heating rate of 3 °C/min and sinusoidal solicitation. The a-PLA (*T*_a_ = 80 °C; *t*_a_ = 1 h) showed a decrease followed by an increase in *E′*, indicating a glass transition followed by a cold crystallization. However, this effect was not as pronounced as in un-PLA due to incomplete crystallization during annealing (${X\prime }_{c}$ = 7.9%). For the other annealed samples, i.e., a-PLA (*T*_a_ = 90–120 °C; *t*_a_ = 1 h), a-PLA (*T*_a_ = 80–120 °C; *t*_a_ = 24 h), and a-PLA/PEG-90/10 (*T*_a_ = 80–120 °C; *t*_a_ = 1 and 24 h), the decline of *E*′ in the glass transition region was significantly reduced, with no further increase, proving that the crystallization was completed during annealing, resulting in semi-crystalline samples [37], which is confirmed by the high ${X\prime }_{c}$ of these samples (Table 5 and Table 6). Effective annealing can improve the heat resistance of PLA by restricting molecular motions and avoiding the drastic drop in *E*′ during the glass transition. However, at high temperatures, *E*′ dropped when the material started to flow due to crystal melting. The test for a-PLA/PEG-90/10 (*T*_a_ = 120 °C; *t*_a_ = 24 h) could not be conducted due to the fragility of the sample, which broke when mounted between the clamps. This sentence indicates that thermal degradation of plasticized PLA is favored by annealing at high temperatures for a long time.

**Figure 17:**
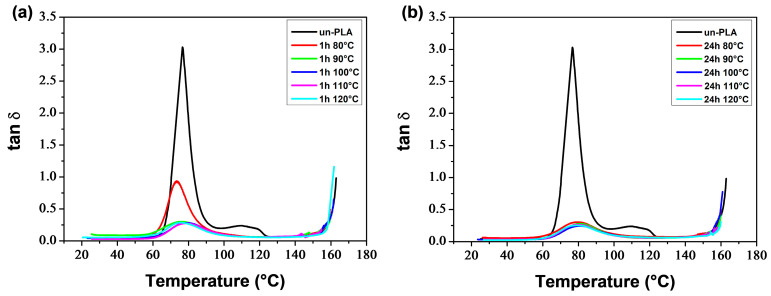
tan *δ* of PLA samples annealed at various temperatures for (**a**) 1 h and (**b**) 24 h.

**Figure 18:**
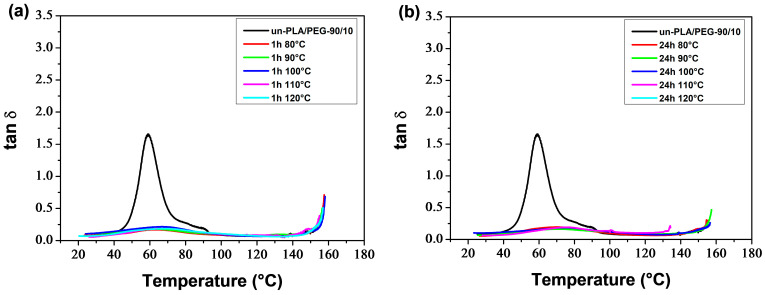
tan *δ* of PLA/PEG-90/10 samples annealed at various temperatures for (**a**) 1 h and (**b**) 24 h.

Figure 17 and Figure 18 show the behavior of tan *δ* for a-PLA and a-PLA/PEG-90/10 samples, respectively. The major relaxation process is associated with the glass transition. Un-PLA and un-PLA/PEG-90/10 exhibited sharp and intense tan *δ* peaks, with maximum values of 3 and 1.6, respectively. This is because in amorphous polymers, there are no restrictions on the motion of the polymer chains. In semi-crystalline polymers, dispersed crystalline regions hinder chain mobility in the amorphous regions, resulting in a reduction of the sharpness and height of the tan *δ* peak [54]. The tan *δ* peak was significantly reduced for a-PLA (*T*_a_ = 80 °C; *t*_a_ = 1 h) with a maximum peak value of 0.9 due to its low crystallinity (${X\prime }_{c}$ = 7.9%) and incomplete crystallization during annealing. The text describes the broad tan *δ* peaks with very low amplitude exhibited by a-PLA (*T*_a_ = 90–120 °C; *t*_a_ = 1 h), a-PLA (*T*_a_ = 80–120 °C; *t*_a_ = 24 h), and a-PLA/PEG-90/10 (*T*_a_ = 80–120 °C; *t*_a_ = 1 and 24 h) due to their semi-crystalline nature (${X\prime }_{c}$ between 23–40%). This behavior is similar to that observed by Tábi et al. [77] for annealed PLA and PLA blended with ethylene vinyl acetate copolymer (PLA/EVA blends). Orue et al. [15] reported a significant reduction in the height of the tan *δ* peak for annealed samples compared to unannealed samples. They found that the tan *δ* peak height of neat PLA, PLA/sisal fiber composites, and PLA/epoxidized oil/sisal fiber systems were considerably reduced after annealing due to the crystallization of PLA.

The DMA thermograms (Figure 15, Figure 16, Figure 17 and Figure 18) can be correlated with the DSC thermograms of Figure 12 and Figure 13, from the perspective of crystalline behavior. The cold crystallization peaks observed on the DSC thermograms for un-PLA, un-PLA/PEG-90/10 and a-PLA (*T*_a_ = 80 °C; *t*_a_ = 1 h), are well correlated with the increase in *E*′ after the glass transition (Figure 15 and Figure 16) for the same samples. This indicates the amorphous state of the unannealed samples (un-PLA and un-PLA/PEG-90/10) and the incomplete crystallization of a-PLA (*T*_a_ = 80 °C; *t*_a_ = 1 h) during annealing. The increase of ${X\prime }_{c}$ and the absence of cold crystallization peaks for a-PLA (*T*_a_ = 90–120 °C; *t*_a_ = 1 h), a-PLA (*T*_a_ = 80–120 °C; *t*_a_ = 24 h), and a-PLA/PEG-90/10 (*T*_a_ = 80–120 °C; *t*_a_ = 1 and 24 h) correlates with a reduction in the decline of *E′* around the glass transition, without any further increase. This reduction is manifested by a strong attenuation of the tan *δ* peak (see Figure 17 and Figure 18).

## 4. Conclusions

This work investigates the effects of plasticization and annealing on PLA properties using DSC, DMA, and rheological measurements. The results show that low molecular weight PEG (*M*_n_ = 400 g/mol) is an efficient plasticizer for PLA. As the PEG content increased, *T*_g_ and *T*_cc_ decreased, while the crystallinity and crystallization rate increased. PLA/PEG blends were found to be miscible up to 20 wt% of PEG content, but a phase separation occurred when the PEG concentration was increased to 30 wt%. The study found that PLA/PEG blends with a maximum of 20 wt% of PEG exhibited a Newtonian plateau at low angular frequencies, followed by shear thinning behavior. The complex viscosity decreased as the plasticizer content increased. The addition of PEG affected both the crystal structure and melting behavior of PLA. The DSC thermograms indicated the formation of a mixture of α′ and α crystals when 1–15 wt% of PEG was added to PLA, as evidenced by the double melting peaks.

Although there has been extensive research on PLA annealing, particularly on α′ and α crystals, little attention has been given to plasticization, with authors often providing only brief descriptions of the double melting peaks [6,14,26,62]. In this study, we closely examine the melting peaks and the α′ and α crystalline forms with varying PEG content and correlate them with the degree of crystallinity. In the concentration range of 1–15 wt% of PEG, the first melting peak decreased as the PEG content increased, while the second peak increased. This indicates a decrease in the α to α′ ratio. Beyond 15 wt% of PEG, only one melting peak was observed, corresponding to the α form. The concentration range of PEG was between 1–15 wt%. This range was characterized by double melting peaks and a significant increase in crystallinity. An inflection point was observed at 15 wt%, after which the crystallinity increased again, but less sharply. Another effect observed in our DMA study is the significant extension of the rubbery plateau towards lower temperatures as a function of PEG content. This is due to crystallites that act as cross-linking nodes.

Neat PLA and PLA/PEG-90/10 samples were annealed at various temperatures (*T*_a_ = 80–120 °C) for durations *t*_a_ of 1 and 24 h. The DSC results indicate that the annealing of PLA samples resulted in the development of the α′ crystalline form at (*T*_a_ = 80 °C; *t*_a_ = 1 and 24 h), both α′ and α forms at (*T*_a_ = 90–110 °C; *t*_a_ = 1 h) and (*T*_a_ = 90–100 °C; *t*_a_ = 24 h), and only the α form at (*T*_a_ = 120 °C; *t*_a_ = 1 h) and (*T*_a_ = 110 and 120 °C; *t*_a_ = 24 h). The addition of PEG altered the crystalline structure of PLA, resulting in a combination of α′ and α crystals, regardless of the annealing time and temperature, except in the case of (*T*_a_ = 120 °C; *t*_a_ = 24 h), where only α crystals were formed. In both the a-PLA and a-PLA/PEG-90/10 samples, the ratio of α to α′ increased with increasing *T*_a_ or *t*_a_. Another important finding is the improvement of crystallinity achieved upon annealing, not only with the increase of *T*_a_ or *t*_a_ but also with the incorporation of PEG. The results of the DMA tests showed that annealing improved the heat resistance of PLA. However, annealing at a high temperature (*T*_a_ = 120 °C) for a long time (*t*_a_ = 24 h) in the presence of PEG resulted in a brittle material, likely due to thermal degradation.

## Figures and Tables

**Table 1:** Thermal characteristics of PLA in PLA/PEG blends, determined from the second heating scan.

PLA/PEG (wt/wt)	*T*_g_ (°C)	*T*_cc_ (°C)	*T*_m1_ (°C)	*T*_m2_ (°C)	Δ*H*_cc_ (J/g)	Δ*H*_m_ (J/g)	*X*_c_ (%)
100/0	59.3	121.5	151.9	—	13.19	13.80	14.8
99/01	56.5	116.0	149.8	153.8	14.07	15.16	16.5
98/02	54.2	113.3	148.9	154.0	14.18	16.21	17.8
95/05	45.9	102.3	148.4	152.0	14.78	17.00	19.2
90/10	32.5	83.7	134.3	149.3	15.52	18.52	22.1
85/15	27.0	77.2	132.9	150.5	13.01	17.58	22.2
80/20	16.5	62.3	147.5	—	9.42	17.59	23.6
70/30	—	76.7	145.0	—	2.31	17.02	26.1

**Table 2:** Kinetic parameters for non-isothermal cold crystallization of PLA/PEG blends.

PLA/PEG (wt/wt)	*t*_1/2_ (min)	*n*	*k*	*k*_c_	*R* ^2^
100/0	2.071	3.34	0.061	0.756	0.9999
99/01	1.938	3.54	0.067	0.763	0.9999
98/02	1.786	3.55	0.088	0.784	0.9999
95/05	1.633	4.12	0.092	0.788	0.9997
90/10	1.354	4.40	0.183	0.844	0.9987
85/15	1.312	4.73	0.192	0.848	0.9983

**Table 3:** Thermo-mechanical properties of neat and plasticized PLA.

PLA/PEG (wt/wt)	*E*_g_ (MPa)	${E\prime }_{\min}$(MPa)	*E*_N_ (MPa)	Δ*T*_N_ (°C)	*T*_α_ (°C)	*T*_cc′_ (°C)
100/0	2428	1.4	107	22.2	76.8	105.0
99/01	2230	2.5	103	25.8	71.8	99.6
98/02	2239	2.2	101	27.0	69.4	98.9
95/05	1727	4.2	92	32.8	64.2	91.2
90/10	1470	5.1	90	41.2	59.1	77.7
85/15	1074	6.5	76	49.8	52.4	64.3
80/20	890	6.7	87	54.5	45.9	56.5
70/30	611	45.3	58	68.2	44.3	53.5

**Table 4:** Parameters of the model of Carreau–Yasuda with yield stress.

PLA/PEG (wt/wt)	*σ*_e_ (Pa)	${\eta *}_{0}$(Pa.s)	*λ* (s)	*n*	*a*	*R* ^2^
100/0	19.5	490	0.0251	0.31983	0.786	0.9995
99/01	12.5	379	0.0186	0.26112	0.798	0.9997
98/02	7.5	214	0.0233	0.32032	0.832	0.9995
95/05	0.7	71	0.0123	0.31301	0.919	0.9995
90/10	0.5	23	0.0046	3.1 × 10^−14^	1.069	0.9909
85/15	0.3	16	0.0045	2.9 × 10^−14^	1.763	0.9676
80/20	0	10	0.0054	5.0 × 10^−1^	4.893	0.8649

**Table 5:** Thermal properties of annealed PLA samples.

*t*_a_ (h)	*T*_a_ (°C)	*T*_g_ (°C)	*T*_cc_ (°C)	Δ*H*_cc_ (J/g)	*T*_exo_ (°C)	*T*_m1_ (°C)	*T*_m2_ (°C)	Δ*H*_m_ (J/g)	${X\prime }_{c}$(%)
-	-	54.2	116.6	16.47	—	149.1	—	17.64	1.3
1	80	52.0	102.9	11.43	135.9	—	152.4	18.78	7.9
1	90	53.4	—	—	—	141.9	152.5	21.36	22.9
1	100	59.6	—	—	—	146.7	153.2	22.97	24.7
1	110	52.4	—	—	—	150.6	—	23.35	25.1
1	120	56.4	—	—	—	150.8	—	24.74	26.6
24	80	51.6	—	—	138.4	—	151.7	17.39	18.7
24	90	55.4	—	—	—	144.8	153.9	22.25	23.9
24	100	56.0	—	—	—	150.0	153.9	24.21	26.0
24	110	57.0	—	—	—	153.1	—	29.39	31.6
24	120	57.5	—	—	—	154.2	—	30.59	32.9

**Table 6:** Thermal properties of annealed PLA/PEG-90/10 samples.

*t*_a_ (h)	*T*_a_ (°C)	*T*_g_ (°C)	*T*_cc_ (°C)	Δ*H*_cc_ (J/g)	*T*_exo_ (°C)	*T*_m1_ (°C)	*T*_m2_ (°C)	Δ*H*_m_ (J/g)	${X\prime }_{c}$(%)
-	-	37.4	82.7	14.89	—	134.2	148.1	17.13	2.7
1	80	40.8	—	—	—	132.2	146.7	22.17	26.5
1	90	40.7	—	—	—	136.1	148.3	23.77	28.4
1	100	41.3	—	—	—	135.3	146.9	25.13	30.0
1	110	40.9	—	—	—	143.0	152.2	24.03	28.7
1	120	36.6	—	—	—	144.1	149.1	25.40	30.3
24	80	41.9	—	—	—	137.3	149.7	28.36	33.8
24	90	41.2	—	—	—	137.2	148.5	28.45	34.0
24	100	48.1	—	—	—	141.7	149.9	29.44	35.1
24	110	43.7	—	—	—	142.3	149.0	32.44	38.7
24	120	43.1	—	—	—	150.1	—	34.45	41.1

## Data Availability

Data set presented in this study is available in this article.
